# Impact of growth parameters on refraction and ocular biometry in Chinese preschool children (3–6 years): the Beijing children eye study

**DOI:** 10.3389/fped.2025.1655087

**Published:** 2025-08-05

**Authors:** Chao Wang, Jing Yuan, Qing Xu, Aimin Jiang

**Affiliations:** Department of Ophthalmology, Beijing Shunyi Hospital, Beijing, China

**Keywords:** growth and development, refraction, ocular biometry, preschool children, axial length

## Abstract

**Background:**

This study aims to investigate the relationship between growth and ocular biometry/refraction in preschool children aged 3–6 years.

**Methods:**

This study performed a retrospective evaluation utilizing anonymized data from a pre-existing cohort of children aged 3–6 years, who were recruited from 11 kindergartens in the Shunyi District of Beijing. Out of the 1,144 children assessed, a total of 1,021 (89.2%) were included in the final analysis. The gathered data adhered to ethical guidelines suitable for subsequent analysis. Information on sex, age, stature, mass, and body mass index (BMI) served as indicators of growth and developmental stages. Ocular measurements such as axial length (AL) and anterior chamber depth (ACD) were obtained using the Lenstar LS900 device. Cycloplegic refraction and corneal curvature were assessed with an autorefractor. The correlation between growth metrics and ocular parameters, including refraction, was examined. To determine statistical significance, regression analyses were carried out.

**Results:**

After making adjustments for age, sex, and weight, every 1-cm increment in height corresponded to a 0.017 mm rise in AL and a 0.005 mm increase in ACD. Regarding weight, following adjustments for age, sex, and height, each 1-kg increase was linked to a 0.017 mm augmentation in AL and a 0.006 mm rise in corneal curvature. Comparable significant correlations were also noted with BMI.

**Conclusion:**

Higher child height is correlated with longer AL, deeper anterior chamber, and flatter cornea. Weight and BMI show similar associations with AL and corneal curvature radius.

## Introduction

The escalating prevalence of myopia in recent years has established it as a critical global public health issue (1, 2). Although corrective lenses can manage refractive error, high or pathological myopia remains an irreversible, sight-threatening condition imposing substantial burdens on individuals and society (2). This is particularly significant in China, where childhood myopia rates continue to rise annually, presenting a major challenge for pediatric eye health (3). While studies report correlations between school-age children's body stature and ocular biometrics or refraction, findings are sometimes inconsistent (1, 2). Preschool children represent a crucial stage of ocular development; however, comprehensive research examining links between their physical growth and ocular biometry remains limited (4, 5). Consequently, extensive investigation into the refractive status of this age group is essential to develop effective myopia prevention and control strategies (6, 7). This large-scale study analyzes data from preschool children, including axial length (AL), anterior chamber depth (ACD), and cycloplegic refraction, to investigate associations between growth parameters, ocular biometrics, and refraction in children aged 3–6 years.

## Methods

### Participants

**Figure 1:**
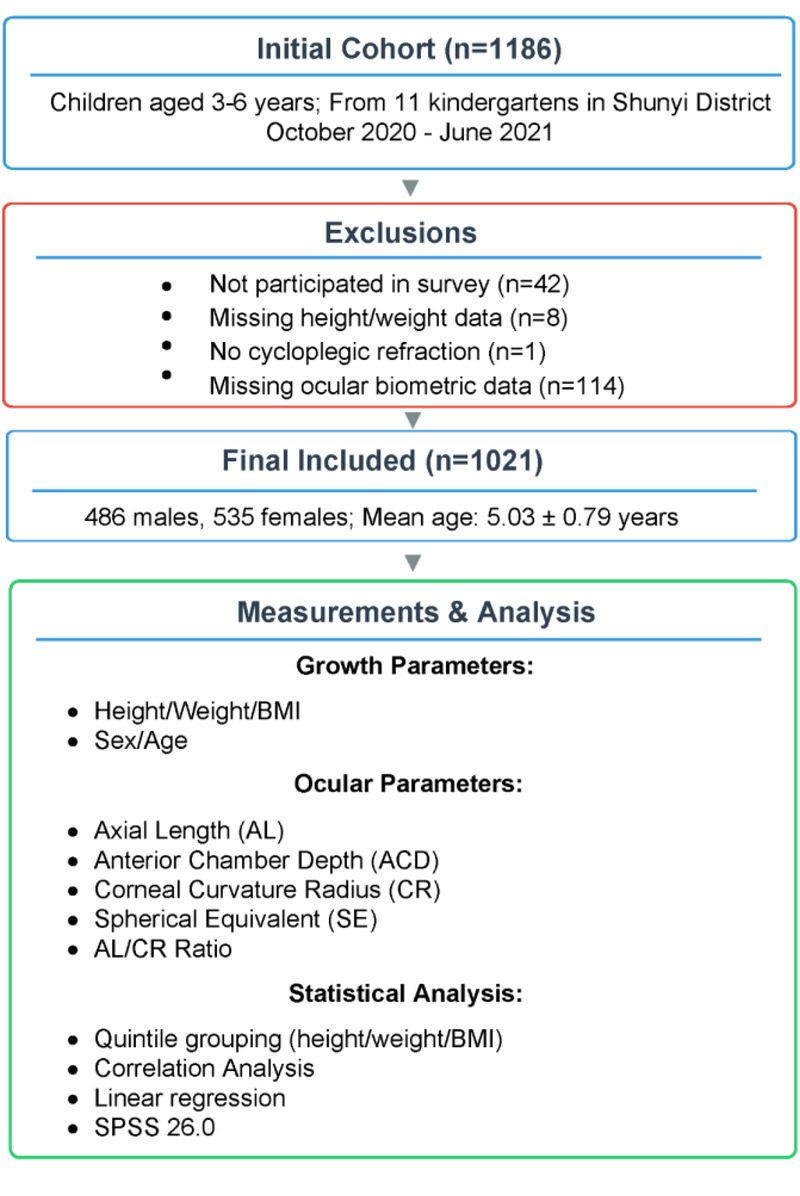
Research and design flowchart.

We retrospectively analyzed data from a cohort of 1,186 children (aged 3–6 years) from 11 kindergartens in Shunyi District, Beijing, who were enrolled between October 2020 and June 2021. Ultimately, 1,144 children participated in the study. Among these 1,144 participants, 1,136 had complete data on height and weight, which were essential for the current analysis. Among the 1,136 participants, one did not undergo cycloplegic refraction, and 114 participants did not provide ocular biometric data. The remaining 1,021 participants (89.2%) were included in the analysis, comprising 486 males and 535 females aged 3–6 years. All children included in the analysis were free of ocular structural abnormalities, Down syndrome, epilepsy, and other psychiatric disorders. The research design flowchart of this study is shown in Figure 1.

Ethical approval for the retrospective analysis of anonymized data was obtained from the Ethics Committee of Shunyi District Hospital, Beijing (Approval No: 2020125). The study adhered to the tenets of the Declaration of Helsinki. The original study obtained written informed consent from parents, and the ethics committee waived the need for additional consent for this secondary analysis.

### Procedures

Basic demographic information of the participating children, such as sex, age, height, and weight, was documented. Standardized protocols were employed across all participants for measuring height and weight. Height was measured with subjects standing barefoot on a height gauge and recorded in centimeters (cm). Weight was determined without shoes or heavy outerwear using a calibrated mechanical scale, and recorded in kilograms (kg). Body mass index (BMI) was calculated using the formula BMI = weight (kg)/height^2^ (m).

Prior to administering the ciliary muscle paralytic agent, ocular biometric parameters, including AL and ACD, were measured using the Lenstar LS900 (Haag-Streit AG, Switzerland). The measurements were repeated three times, and the average values were recorded. A 1% cyclopentolate solution (Alcon, USA) was used as the ciliary muscle paralytic agent. One drop was instilled every 5 min, totaling three drops. After 30 min, ciliary muscle paralysis was determined based on pupil light reflex. Ciliary muscle paralysis was defined as the disappearance of the pupil light reflex with a pupil diameter greater than 6 mm. If the pupil light reflex persisted after the last instillation for 30 min, additional 1% cyclopentolate drops were administered until the pupil light reflex disappeared. Subsequently, objective cycloplegic refraction was performed by an autorefractor (ARK-1, NIDEK, Japan) to examine spherical equivalent (SE) and corneal curvature radius (CR), with three measurements taken and averaged. The AL/CR ratio was defined as AL divided by the mean CR (This ratio is a known predictor of myopia risk and reflects the overall eye shape, independent of the individual AL and CR values). Myopia was defined as SE ≤−0.5 diopters (D), mild hyperopia as 0.50 D ≤ SE < 2.00 D, and mild-to-high hyperopia as SE ≥+2.00 D. Children with hyperopia ≤0.50 D and >−0.50 D were classified as emmetropic; recent evidence suggests this group includes “premyopia”—a high-risk state for future myopia onset (8)—but formal premyopia stratification was not employed here due to diagnostic uncertainty in preschoolers.

All examinations were conducted by ophthalmologists and optometrists who underwent uniform training. Relevant personnel received training, and standardized criteria and procedures were established. Supervisors were appointed to periodically oversee the examination processes. All children underwent examinations using the same instrument, with regular maintenance and calibration of the relevant equipment. A data administrator was assigned to cross-verify information with ophthalmologists after each examination, promptly identifying and rectifying any omissions or errors. Data entry was carried out independently by two individuals using Epidata 3.1 software, and after ensuring consistency, the data were archived in the database.

### Statistical analyses

Statistical analysis was executed using SPSS 26.0 software (SPSS, Chicago, IL, USA). Given the high correlation between both eyes for refraction and ocular biometrics (Pearson correlation coefficients: SE 0.86; AL 0.967; ACD 0.877; CR 0.929; AL/CR 0.817; *p* < 0.001), data from the right eye were selected for analysis. Height, weight, and BMI were categorized into quintiles, and the median of each height category was considered a continuous variable for linear trend tests to assess significance (9). The normality of all continuous outcome parameters (e.g., AL, ACD, CR, SE) was assessed using the Shapiro–Wilk test before further analyses.

Univariate linear regression models were used to explore the relationships between height, weight, BMI, and various ocular biometric parameters or refraction. Multivariate linear regression models adjusted for age, sex, height, or weight were constructed to evaluate the impact of height, weight, and BMI (independent variables) on ocular biometric parameters or refraction (dependent variables). These measures (age, height, weight, SE, AL, ACD, CR) are reported as mean ± SD. A two-sided *p*-value less than 0.05 was deemed statistically significant.

## Results

### Study population characteristics

This study analyzed data from 1,021 children aged between 3 and 6 years old. Among these participants, 47.6% were male and 52.4% female (refer to Table 1 for detailed demographics). The average age was calculated at 5.03 ± 0.79 years, with an average height of 112.52 ± 7.17 cm, and a mean weight of 20.62 ± 4.66 kg. Other notable measurements include an average AL of 22.28 ± 0.67 mm, ACD of 3.31 ± 0.25 mm, corneal curvature radius of 7.75 ± 0.26 mm, and a mean refraction value of 1.20 ± 0.80 diopters. Myopia was present in 3.13% (32 out of 1,021) of the subjects, while mild hyperopia was observed in 73.07% (746 out of 1,021), and moderate to severe hyperopia accounted for 15.87% (162 out of 1,021).

**Table 1:** Characteristics of the study population.

Characteristic	Value
Total participants	1,021
Males [*n* (%)]	486 (47.6%)
Females [*n* (%)]	535 (52.4%)
Age (years)	5.03 ± 0.79
Height (cm)	112.52 ± 7.17
Weight (kg)	20.62 ± 4.66
AL (mm)	22.28 ± 0.67
ACD (mm)	3.31 ± 0.25
CR (mm)	7.75 ± 0.26
SE(D)	1.20 ± 0.80
Myopia [*n* (%)]	32 (3.13%)
Mild hyperopia [*n* (%)]	746 (73.07%)
Mild-to-high hyperopia [*n* (%)]	162 (15.87%)

AL, axial length; ACD, anterior chamber depth; CR, corneal curvature; AL/CR, axial length-corneal radius; SE, spherical equivale.

### Quintile analysis of ocular parameters

**Figure 2:**
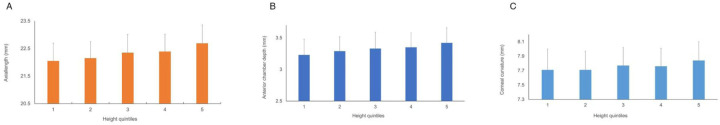
Ocular biometric parameters stratified by height quintiles. **(A)** Axial length (AL), **(B)** anterior chamber depth (ACD), and **(C)** corneal curvature radius (CR). Values represent means. Trend analysis used linear regression with median quintile height as a continuous variable. Children in the highest height quintile (Q5) showed significantly longer AL, deeper ACD, and flatter CR vs. Q1.

Table 2 outlines the mean values of refraction and ocular biometrics across quintiles defined by height, weight, and BMI. No significant associations were found between height or BMI and refraction levels. Generally, taller and heavier children displayed longer axial lengths, deeper anterior chambers, flatter corneas, and higher AL/CR ratios. Specifically, compared to those in the lowest quintile for height, children in the highest quintile showed increases of 0.64 mm in AL, 0.19 mm in ACD, 0.13 mm in corneal curvature radius, and a 0.03 increase in the AL/CR ratio. Figures 2A–C visually represent these trends for height categories.

**Table 2:** Mean ocular biometry measurements and refraction by quintiles of stature.

Stature	*n*	Median	Ocular biometry				Refraction
			AL (mm)	ACD (mm)	CR (mm)	AL/CR	SE(D)
Height (m)							
1st quintile	208	1.04	22.05 ± 0.65	3.23 ± 0.25	7.71 ± 0.29	2.86 ± 0.07	1.23 ± 0.78
2nd quintile	271	1.10	22.15 ± 0.60	3.29 ± 0.23	7.71 ± 0.26	2.87 ± 0.07	1.22 ± 0.81
3rd quintile	223	1.14	22.35 ± 0.66	3.33 ± 0.26	7.77 ± 0.25	2.88 ± 0.06	1.18 ± 0.84
4th quintile	200	1.18	22.39 ± 0.63	3.35 ± 0.23	7.76 ± 0.25	2.88 ± 0.06	1.25 ± 0.81
5th quintile	119	1.25	22.69 ± 0.67	3.42 ± 0.24	7.84 ± 0.26	2.89 ± 0.06	1.03 ± 0.72
*p* (trend)			*p* < 0.001	*p* < 0.001	*p* < 0.001	*p* < 0.001	*p* = 0.162
Weight (kg)							
1st quintile	227	16.0	22.04 ± 0.61	3.25 ± 0.24	7.70 ± 0.27	2.86 ± 0.07	1.22 ± 0.77
2nd quintile	222	18.0	22.17 ± 0.62	3.30 ± 0.25	7.72 ± 0.27	2.87 ± 0.06	1.25 ± 0.75
3rd quintile	178	20.0	22.32 ± 0.66	3.32 ± 0.24	7.76 ± 0.24	2.88 ± 0.06	1.20 ± 0.77
4th quintile	194	22.0	22.35 ± 0.64	3.35 ± 0.25	7.77 ± 0.25	2.88 ± 0.07	1.28 ± 0.85
5th quintile	200	26.0	22.58 ± 0.69	3.36 ± 0.25	7.82 ± 0.27	2.89 ± 0.07	1.02 ± 0.85
*p* (trend)			*p* < 0.001	*p* < 0.001	*p* < 0.001	*p* = 0.005	*p* = 0.015
BMI (kg/m^2^)							
1st quintile	210	13.72	22.21 ± 0.63	3.32 ± 0.26	7.72 ± 0.26	2.88 ± 0.06	1.21 ± 0.71
2nd quintile	205	14.85	22.14 ± 0.64	3.29 ± 0.25	7.69 ± 0.26	2.88 ± 0.07	1.20 ± 0.75
3rd quintile	198	15.66	22.30 ± 0.61	3.30 ± 0.25	7.78 ± 0.25	2.87 ± 0.07	1.30 ± 0.75
4th quintile	205	16.80	22.33 ± 0.72	3.31 ± 0.25	7.77 ± 0.28	2.88 ± 0.07	1.19 ± 0.93
5th quintile	203	19.17	22.44 ± 0.69	3.35 ± 0.25	7.79 ± 0.27	2.88 ± 0.07	1.08 ± 0.83
*p* (trend)			*p* < 0.001	*p* = 0.167	*p* < 0.001	*p* = 0.364	*p* = 0.109

Children were categorized into quintiles (Q1–Q5) based on height, weight, and BMI. Ocular parameters are reported as median (IQR). The *p*-value for trend was calculated using linear regression with the median value of each quintile as a continuous variable. BMI, body mass index; AL, axial length; ACD, anterior chamber depth; CR, corneal curvature; AL/CR, axial length-corneal radius; SE, spherical equivale.

### Correlation analysis

The correlations among height, weight, BMI, gender, age, refraction, and ocular biometric parameters are summarized in Table 3. Both height and weight showed strong positive correlations with age, moderate positive correlations with AL, and weak positive correlations with ACD, CR, and AL/CR. Negative correlations with female gender and refraction (SE) were weak. BMI exhibited weak positive correlations with AL and CR and negligible correlations with other parameters.

**Table 3:** Correlation analysis between body stature and refraction and ocular biometry.

Variable	Spearman correlation co-efficient		
	Height (cm)	Weight (kg)	BMI
Age	0.700**	0.451**	−0.023
Sex (female)	−0.062*	−0.075**	−0.053*
AL (mm)	0.300**	0.281**	0.137**
ACD (mm)	0.220**	0.168**	0.041
CR (mm)	0.139**	0.171**	0.131**
AL/CR	0.193**	0.132**	0.011
SE(D)	−0.072*	−0.090**	−0.067*

ACD, anterior chamber depth; AL, axial length; CR, corneal curvature; AL/CR, axial length-corneal radius; SE, spherical equivalent.

* *p*-value <0.05.

** *p*-value <0.01.

### Linear regression analysis

**Figure 3:**
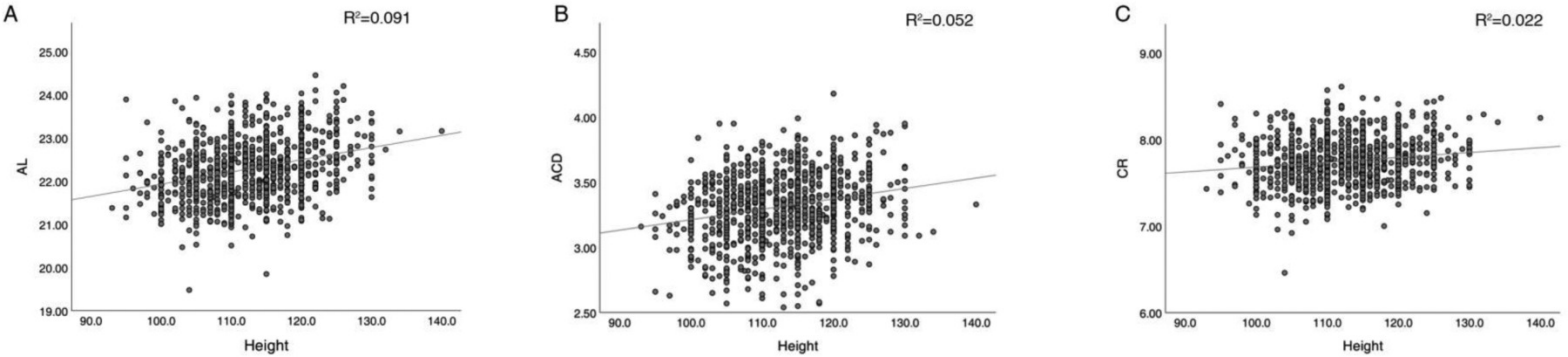
Scatterplots of height vs. ocular biometric parameters with regression lines. **(A)** Axial length (AL), **(B)** anterior chamber depth (ACD), **(C)** corneal curvature radius (CR). Solid lines indicate linear regression fits; shaded areas represent 95% confidence intervals. Height was positively correlated with AL, ACD, and CR in univariate analysis.

Results from the regression analyses are presented in Table 4. Each entry reflects univariate linear regression findings, with refraction or ocular biometrics as dependent variables and height, weight, or BMI as independent variables, adjusted for other covariates. An increase of 1 cm in height was associated with increments of 0.028 mm in AL, 0.008 mm in ACD, 0.006 mm in corneal curvature radius, and a 0.002 unit rise in the AL/CR ratio. Weight and BMI similarly influenced various ocular parameters, albeit without significant correlation to spherical equivalent (SE). Scatterplots (Figures 3A–C) further illustrate these relationships.

**Table 4:** Results of linear regression analysis of growth and development parameters with ocular biometric parameters.

Variable	Crude data	*p*	R^2^	Model 1	*p*	R^2^	Model 2^a^	*p*	R^2^ final
Height (cm)									
AL (mm)	0.028 (0.023, 0.034)	<0.001	0.091	0.024 (0.017, 0.032)	<0.001	0.215	0.017 (0.009, 0.026)	<0.001	0.222
ACD (mm)	0.008 (0.006, 0.010)	<0.001	0.052	0.005 (0.002, 0.008)	0.001	0.117	0.005 (0.001, 0.008)	0.007	0.116
CR (mm)	0.006 (0.003, 0.008)	<0.001	0.022	0.009 (0.006, −0.012)	<0.001	0.085	0.007 (0.003, 0.010)	<0.001	0.090
SE(D)	−0.005 (−0.012, 0.002)	0.135	0.002	−0.01 (−0.020, −0.001)	0.036	0.003	−0.006 (−0.018, 0.005)	0.272	0.004
AL/CR	0.002 (0.001, 0.002)	<0.001	0.027	0.0002 (−0.001, 0.001)	0.599	0.073	0.0002 (−0.001, 0.001)	0.591	0.072
Weight (kg)									
AL (mm)	0.039 (0.030, 0.047)	<0.001	0.072	0.027 (0.018, 0.035)	<0.001	0.211	0.017 (0.007, 0.026)	0.007	0.222
ACD (mm)	0.008 (0.005, 0.011)	<0.001	0.021	0.003 (−0.0004, 0.0063)	0.085	0.110	0.0002 (−0.004, 0.004)	0.919	0.116
CR (mm)	0.01 (0.006, 0.013)	<0.001	0.028	0.01 (0.006, 0.013)	<0.001	0.080	0.006 (0.001, 0.01)	0.009	0.090
SE(D)	−0.012 (−0.022, −0.001)	0.032	0.004	−0.013 (−0.025, −0.002)	0.024	0.004	−0.009 (−0.023, 0.004)	0.167	0.004
AL/CR	0.001 (0.001, 0.002)	0.001	0.009	−0.00006 (−0.001, 0.001)	0.900	0.072	0.000089 (−0.001, 0.001)	0.867	0.072
BMI (kg/m^2^)									
AL (mm)	0.028 (0.014, 0.042)	<0.001	0.013	0.025 (0.012, 0.038)	<0.001				0.192
ACD (mm)	0.001 (−0.004, 0.007)	0.665	−0.001	0.001 (−0.005, 0.006)	0.824				0.108
CR (mm)	0.01 (0.004, 0.016)	0.001	0.011	0.009 (0.003, 0.014)	0.002				0.065
SE(D)	−0.015 (−0.032, 0.002)	0.092	0.002	−0.014 (−0.032, 0.003)	0.105				0.001
AL/CR	−0.00005 (−0.001, 0.001)	0.948	−0.001	−0.00004 (−0.001, 0.001)	0.96				0.072

Data in parentheses represent the 95% confidence interval. All regression co-efficients are derived from a separate regression model with the individual refraction or ocular biometric components as the dependent variable, and height, weight, or BMI as the independent variable, adjusting for other covariates. Model 1 is adjusted for age and gender, Model 2 is adjusted for age, gender, and weight or height. ACD, anterior chamber depth; AL/CR, axial length-corneal radius; AL, axial length; BMI, body mass index; CR, corneal curvature; SE, spherical equivalent.

^a^ Models for height are adjusted for weight, and vice versa. Models for BMI are not adjusted for either height or weight.

In multivariate analysis, after adjustments for age, gender, and weight, height remained significantly associated with changes in AL, ACD, and corneal curvature radius, whereas no such associations were evident for SE or the AL/CR ratio. Similar patterns emerged for weight and BMI, influencing specific ocular dimensions without affecting SE or the AL/CR ratio significantly. The model's adequacy was confirmed through residual vs. fitted values scatterplots.

## Discussion

This retrospective study analyzed a large-scale dataset of preschool children, including AL, ACD, and cycloplegic refraction measurements, to examine associations between growth/developmental parameters and refractive/ocular biometric outcomes. Multivariable analysis, adjusting for age, gender, and weight, demonstrated that increased height was associated with greater AL, deeper ACD, and flatter corneal curvature. Furthermore, after controlling for age, gender, and height, increased body weight was associated with greater AL and flatter corneal curvature.

Increases in average height and weight over recent decades have paralleled a rise in myopia prevalence, suggesting a shared underlying mechanism (10–12). However, consensus on the correlation between height and refractive error remains elusive. A survey conducted by Ye et al. (13) on children aged 6–15 in Tianjin, after controlling for covariates, indicated a correlation between height and AL, inferring a potential link between refractive development and physical growth in children. In a school-based study of 7–9-year-old Singaporean Chinese children, Saw et al. (14) found that taller children in Singapore had a higher prevalence of myopia and more negative refractive errors. However, other studies have suggested no correlation between refractive error and height (15, 16). Few studies have been conducted in children aged 3–6 years, who are in a critical period of vision development, and in particular, the measurement of refractive parameters after ciliary muscle paralysis is more difficult to carry out due to the poor cooperation of children and parents (9, 17). Building on previous work, this study offers a more detailed analysis. In this study, univariate analysis suggested a negative correlation between height and refraction. However, after adjusting for age, sex, and weight, no significant correlation was observed. This may be due to that refraction is influenced by various factors in children, including physical development, genetic factors, family economic status, near-work time, outdoor activities, and visual habits (18–20). Thus, there may not be a direct relationship between height and refraction.

Prior research has established a link between height and AL, providing indirect or direct evidence for coordinated growth between the eyes and the body. Taller individuals often exhibit deeper anterior chambers, longer axial lengths and flatter corneas (21, 22). In this study, univariate analysis and quintile grouping based on height revealed this association. Importantly, even after adjusting for age, sex, and weight, this relationship persisted, indicating that children with greater height tend to have larger overall eye dimensions. Hence, we posit that ocular development may proceed concurrently with the overall growth and development of children. The correlation between height and AL has been reinforced by recent longitudinal studies. Chen et al. (23) demonstrated that height growth and AL elongation are significantly synchronized before age 12 in non-myopic children, suggesting body development drives ocular expansion during early growth phases. Similarly, Li et al. (24) confirmed through multivariate regression that AL maintains a significant positive association with height in Chinese preschoolers after controlling for confounders.

Moreover, earlier studies have indicated that the height of Chinese adolescents correlates with the AL/CR ratio, which reflects the overall eye size (25, 26). Nonetheless, the literature presents conflicting findings, with some investigations proposing no connection between height and the AL/CR ratio (24). In our univariate linear regression analysis, we identified a positive correlation between height and the AL/CR ratio. However, in the multivariate linear regression adjusting for confounding factors, this correlation was not significant. The conflicting results may be attributed to variations in age, refractive status, sample size, and the lack of control for other confounding factors in the studies (27, 28).

The refractive condition is physiologically governed by biometric parameters like AL, CR, ACD, and lens thickness (LT) (9). It is commonly accepted that a reduction in corneal curvature can offset the elongation of AL to keep images sharply focused on the retina (29). Myopia usually arises in an eye that has become too long. If AL increases beyond the total refraction of the cornea and lens, it can lead to Myopia typically develops in eyes where the AL has extended excessively. When AL surpasses the total refractive capacity of the cornea and lens, myopia may occur. In this research, height demonstrated a strong association with AL, indicating a possible shared mechanism between height and ocular growth. As Fuse et al. (30) have shown, the significant correlation between AL and height can be attributed to common genetic factors.

Compared to height, the correlation between weight, BMI, and refractive parameters has been less studied. Our findings show similarities between the relationships of weight/BMI and ocular biometric parameters and those observed with height. The economic development in China has led to increased consumption of foods high in saturated fats and cholesterol, which has been associated with longer AL in children (31, 32).

Regarding ocular biometric parameters, this research revealed a notable relationship between body weight, BMI, and AL, CR. Interestingly, no significant associations were observed between BMI and ACD or SE. This lack of correlation may reflect distinct physiological pathways: ACD is primarily influenced by anterior segment development, which appears less responsive to systemic metabolic factors represented by BMI. Similarly, SE's multifactorial nature—modulated by genetic, environmental, and compensatory mechanisms (e.g., lens thinning)—could dilute BMI-specific effects. Prior study support this dissociation; Wu et al. (33) reported no BMI-ACD linkage. It should be noted that the r^2^ values from the final regression analysis were comparatively low. The interaction between weight, BMI, refractive error, and ocular biometrics seems complex, and these observed relationships may introduce additional confounding factors.

This study has several strengths, including the standardized measurement of refractive error after ciliary muscle paralysis and the use of standardized protocols for measuring ocular biometric data. Additionally, the large sample size and control for potential confounding factors are notable advantages, addressing gaps identified in previous reports. In this study, cycloplegic refraction was performed in children aged 3–6 years to establish a detailed refractive profile, which serves as a critical foundation for early myopia prevention strategies. The preschool age range (3–6 years) is particularly significant because it represents a crucial period during which ocular growth patterns are established and can be influenced by various environmental and genetic factors (27, 28). Despite the challenges associated with conducting such assessments in young children, addressing this age group is essential due to its potential impact on long-term ocular health outcomes. By focusing on this understudied age range, our research provides foundational data that can help predict the onset of myopia in Chinese school-age children and inform targeted public health interventions aimed at mitigating the burden of myopia. Our findings suggest that early detection and monitoring of ocular biometric parameters in preschool-aged children could play a pivotal role in developing effective preventive measures.

Despite the strengths of this study, including a large sample size and standardized protocols, it is important to note that the cross-sectional design limits causal inference. Future longitudinal studies are needed to better understand the temporal relationship between growth parameters and ocular biometrics. Furthermore, cooperation issues during cycloplegic refraction (e.g., head movement, fixation instability) may introduce greater error margins in SE and ACD measurements vs. older cohorts, potentially attenuating true associations. Another limitation of this study is that several important potential confounders known to affect myopia risk were not controlled for, including genetic predisposition, screen time, time spent outdoors, parental myopia, and socioeconomic status. These factors could influence the observed associations and should be considered in future research. Moreover, we did not formally stratify children with low hyperopia (emmetropic range: SE ≤0.50 D and >−0.50 D), who may represent a “premyopia” subgroup at high risk for future myopia onset, potentially underidentifying at-risk children within this group. Additionally, BMI values were not adjusted for age using percentiles or Z-scores, which may limit their interpretability in young children. Future studies should consider normalizing BMI values to account for age-specific variations, thereby enhancing the accuracy of the findings.

## Conclusion

After controlling for potential confounding factors, it was observed that taller children had longer eye axial lengths, deeper anterior chambers, and flatter corneas. Similar correlations were found between weight, BMI, and eye AL, as well as corneal curvature radius. These differences in height and weight may partially explain variations in refractive error and ocular biometric parameters.

## Data Availability

The raw data supporting the conclusions of this article will be made available by the authors, without undue reservation.
